# Pictorial Review of Soft Tissue Lesions with Calcification

**DOI:** 10.3390/diagnostics15070811

**Published:** 2025-03-22

**Authors:** Zahra Masroori, Peyman Mirghaderi, Sara Haseli, Arash Azhideh, Bahar Mansoori, Eric Chen, Chankue Park, Majid Chalian

**Affiliations:** 1 Department of Radiology, Division of Musculoskeletal Imaging and Intervention, University of Washington, Seattle, WA 98105, USAmchalian@uw.edu (M.C.); 2 Department of Radiology, Division of Abdominal Imaging, University of Washington, Seattle, WA 98105, USA

**Keywords:** soft tissue tumors, radiologic findings, soft tissue calcification

## Abstract

Calcifications in soft tissue tumors present critical diagnostic challenges in musculoskeletal imaging. Their presence and morphology can provide key clues for differentiating benign from malignant lesions, influencing both prognosis and management strategies. This pictorial review aims to explore the imaging characteristics, patterns, and implications of soft tissue calcifications, with a focus on distinguishing between benign and malignant soft tissue tumors based on the World Health Organization classification. A systematic evaluation of imaging findings in various soft tissue tumor subtypes, including adipocytic, smooth muscle, vascular, chondro-osseous, and tumors of uncertain differentiation, is presented. Additionally, non-neoplastic causes of soft tissue calcifications, such as metabolic and inflammatory conditions, are reviewed for comprehensive differential diagnosis. Our review shows that the presence, distribution, and morphology of calcifications, such as stippled, punctate, coarse, and amorphous patterns, play a crucial role in tumor characterization. Some important examples are phleboliths, which strongly suggest a benign hemangioma, while dystrophic calcification is more commonly associated with malignant entities such as synovial sarcoma and dedifferentiated liposarcoma. Peripheral calcifications with zonal distribution are characteristic of myositis ossificans, whereas central dense calcifications may indicate extra-skeletal osteosarcoma. The review also discusses the significance of calcifications in non-neoplastic conditions, such as calcific tendinitis, tumoral calcinosis, and metabolic diseases, which can mimic soft tissue tumors. Recognizing the imaging characteristics of soft tissue calcifications is essential for accurate tumor classification and appropriate clinical management. This review highlights the importance of integrating radiologic findings with clinical and histopathological data to avoid misdiagnosis and unnecessary interventions.

## 1. Introduction

**Figure 1:**
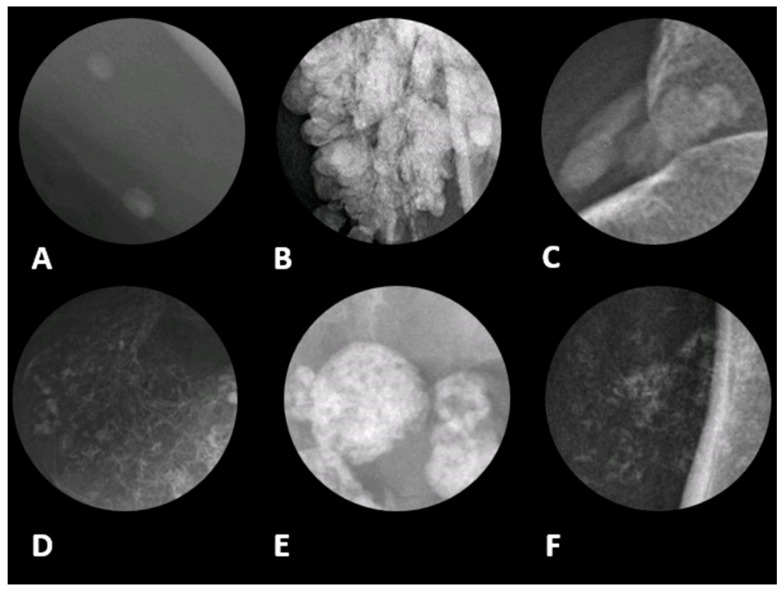
Different radiographic patterns of calcification in soft tissue with associated differential diagnoses in magnified radiographic views. (**A**) Phleboliths, often appearing as oval or round calcifications and are commonly associated with venous malformations and hemangiomas. It typically indicates a benign etiology. (**B**) Globular pattern refers to large, lobulated calcifications resembling clusters, seen in tumoral calcinosis, chronic renal failure, and calcinosis universalis. It is likely a benign etiology; however, the exact diagnosis depends on location and clinical history. (**C**) Amorphous, which is characterized by irregular calcifications without a distinct shape. This pattern is typically seen in dystrophic calcifications resulting from tissue damage, such as calcified tendinitis. (**D**) Chondroid pattern with rings and arcs calcifications, suggesting a cartilaginous origin. This is characteristic of chondrosarcoma, chondromas, and synovial chondromatosis. (**E**) Coarse pattern can be found in damaged or necrotic tissues, including certain types of soft tissue sarcomas (**F**) Stippled or punctate pattern, which appear as multiple small, dot-like calcifications. This pattern can be seen in both benign and malignant tumors. The image was obtained from the University of Washington Medical Imaging database with the required permissions.

Differentiating soft tissue tumors as benign versus malignant is challenging, and various imaging methods play a crucial role in this process [1,2]. Soft tissue tumors are especially challenging to diagnose as the World Health Organization (WHO) recognizes over 100 types, many of which have similar features in pathology and imaging [3,4]. Various imaging features are used to evaluate whether a tumor is benign or malignant, including its location, shape, signal intensity, pattern of enhancement, and presence of fat, hemorrhage, osseous destruction, and calcifications. These radiologic features help predict prognosis and guide early treatment in malignant tumors, while preventing unnecessary procedures in benign tumors [5]. Presence of calcification, its distribution within the lesion (e.g., central, peripheral), and morphology (e.g., amorphous, spiculated, coarse) (Figure 1) are useful in determining malignancy [5,6,7].

The authors present a review on calcifications in evaluating the malignancy of a mass, organized by soft tissue tumor subtype as classified by the WHO [4]. The tumors discussed are divided into adipocytic tumors, smooth muscle tumors, vascular tumors, chondro-osseous tumors, and tumors of uncertain differentiation.

## 2. Overview of Soft Tissue Tumors Containing Calcifications

Soft tissue tumors are classified into 12 distinct categories according to the 2020 WHO classification, which groups them based on histogenesis and molecular characteristics. This classification system includes benign, intermediate (locally aggressive or rarely metastasizing), and malignant tumors [4].

Soft tissue tumors with calcifications are shown in Table 1.

If soft tissue calcifications are present but the presence of a tumor is unlikely, non-neoplastic soft tissue calcifications should be considered. These may include calcific tendinitis, metabolic disease (gout, tumoral calcinosis, chronic renal failure), pilomatricoma, synovial chondromatosis, calcific myonecrosis, injection granuloma, cysticercosis, calcinosis cutis, and others, which can be differentiated based on clinical information and imaging patterns (Table 2).

## 3. Radiological Manifestations of Calcifications

### 3.1. Etiology

Soft tissue calcifications are traditionally categorized by etiology: dystrophic, metabolic (metastatic), and iatrogenic [8,9]. Dystrophic calcification refers to calcium salt precipitation secondary to the alkaline cellular environment of damaged necrotic soft tissue. Metabolic (metastatic) calcification describes calcium deposition in normal tissue due to altered metabolic conditions (e.g., hypercalcemia, hyperphosphatemia). Iatrogenic calcifications are caused by medical interventions such as injections [8].

### 3.2. Morphology

Osseous calcification refers to dense calcifications with bony trabeculae; these calcifications can be distributed in a peripheral zonal pattern or a central pattern [10]. The peripheral zonal pattern of shell-like calcification in a soft tissue lesion is characteristic of myositis ossificans. On the other hand, a soft tissue mass with a central pattern of dense osseous calcifications is more suggestive of extra-skeletal osteosarcoma [11].

Chondroid calcifications have a pattern of cartilaginous matrix and typically appear as curvilinear, “popcorn”, or “rings and arcs” calcifications [12]. They are inconclusive in determining whether a mass is benign or malignant.

Phleboliths are round, lamellated calcifications with a central lucency. A soft tissue mass with multiple phleboliths is strongly suggestive of hemangioma [10] and malignancy is exceedingly rare.

Other patterns of intra-tumoral calcifications include stippled, punctate, coarse, and amorphous calcifications. Stippled, punctate, and coarse calcifications are non-specific patterns that are seen in both benign and malignant tumors. It is difficult to distinguish the pattern depending on the size of the calcification. Amorphous calcifications can be described as cloud-like and poorly defined, and are more typically seen in non-neoplastic soft tissue lesions such as calcific tendinitis or metabolic diseases [10,13]. Less commonly, amorphous calcifications happen in malignant sarcomas, including synovial sarcomas, liposarcomas, and leiomyosarcomas [14,15].

## 4. Soft Tissue Tumors with Calcifications According to Cell-Type

### 4.1. Adipocytic Tumors

**Figure 2:**
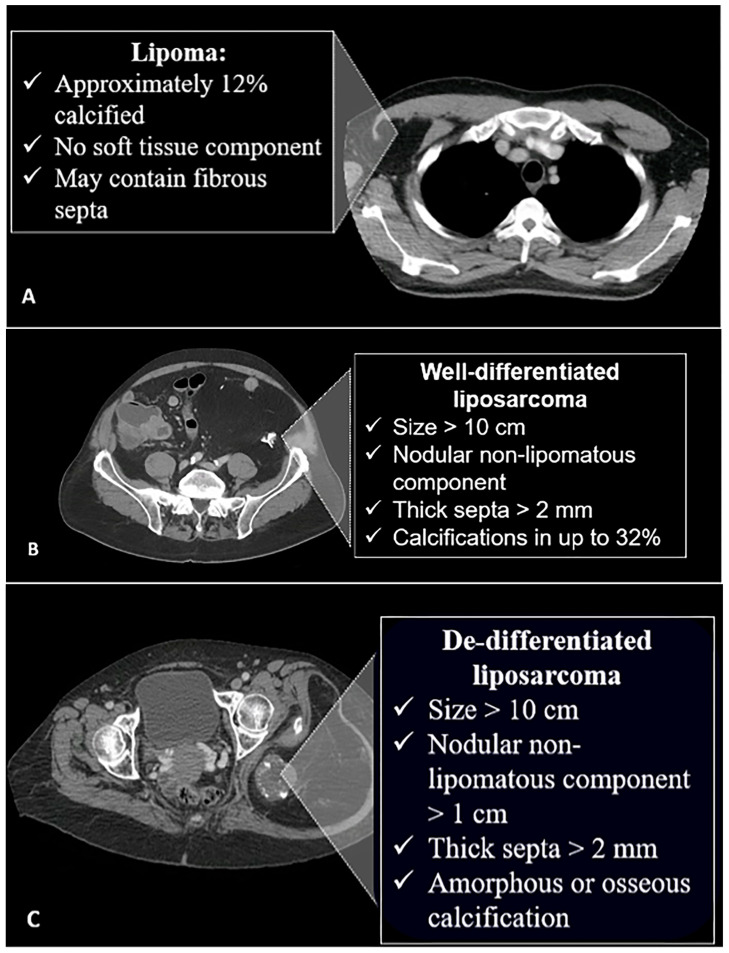
Characteristic features of different fat-containing lesions on CT scan. (**A**) Lipomas may contain septa and might have calcifications in 12% of cases. (**B**) Well-differentiated liposarcomas are larger, with non-lipomatous nodular components and thickened septa. Calcifications are more common and seen in 32% of cases. (**C**) In the de-differentiated form, the lesions are larger and may contain amorphous or osseous calcifications. The image was obtained from the University of Washington Medical Imaging database with the required permissions.

While calcifications in adipocytic tumors are not specific, malignant adipocytic tumors are more likely to show calcifications than benign ones, and studies have reported calcifications in approximately 32% of liposarcomas and 11% of lipomas [5,6]. These calcifications are most commonly coarse [7,16] due to fat necrosis [17]. At imaging, lipoma and liposarcoma may share a similar characteristic appearance as a mass predominantly composed of highly differentiated adipose tissue. Imaging signs that suggest a malignant liposarcoma rather than a benign lipoma (Figure 2) include the presence of thick septa, a size larger than 10 cm, nodular non-lipomatous components, and a composition of less than 75% fat [6].

**Figure 3:**
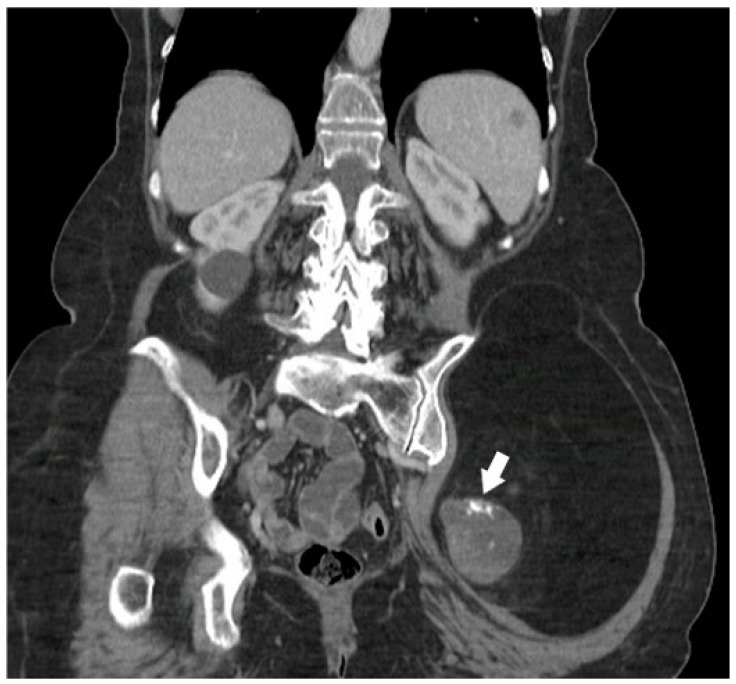
A 68-year-old female with a palpable firm left gluteal mass on physical exam. Coronal CT demonstrates thick nodular non-lipomatous area >1 cm (arrow) and peripheral calcifications. The histopathology result was in favor of de-differentiated liposarcoma. The image was obtained from the University of Washington Medical Imaging database with the required permissions.

Liposarcoma comprises two components: well-differentiated liposarcoma-type component and undifferentiated sarcoma-type cells. The latter manifests as collagenized tissues, metaplastic mineralization, or fat necrosis. In a case report of dedifferentiated liposarcoma, amorphous or osseous calcifications were most dense in the center, similar to mineralization patterns of extra-osseous osteosarcomas [17]. Other features (Figure 3) suggestive of dedifferentiated liposarcoma include a larger non-lipomatous component, especially a nodular non-lipomatous area larger than 1 cm [7,18].

### 4.2. Fibroblastic/Myofibroblastic Tumors

Peripheral osseous calcifications in a zonal distribution are strongly suggestive of myositis ossificans, especially with a history of trauma, burns, arthroplasty, and paraplegia [12,19]. Myositis ossificans grows rapidly in the first four weeks. Histologically, it exhibits a zonal pattern with a central area of proliferating fibroblasts and a peripheral zone of mature bone. Peripheral calcifications are detectable by radiograph at 4-10 weeks. CT and MRI can detect calcifications even earlier than radiographs. If the characteristic imaging features are present, myositis ossificans can be followed by imaging instead of biopsy or surgery. The lesion often resolves spontaneously [2,20]. After 6 months of maturation, these characteristic features include a well-defined peripheral cortex and internal trabeculae [19].

### 4.3. Calcifying Aponeurotic Fibroma

Calcific aponeurotic fibroma is a rare benign tumor that affects fascia, tendon, and aponeurosis of extremities in young individuals [21]. Despite its benign nature, it can become locally invasive, making it difficult to distinguish from a malignant tumor [22]. The characteristic radiologic presentation includes intralesional calcifications on plain radiographs and a poorly defined mass on MRI, with hyposignal intensity on T1-weighted images and variable signal intensity on T2-weighted images. Calcifications may appear as areas of low signal intensity [23].

### 4.4. Vascular Tumors

**Figure 4:**
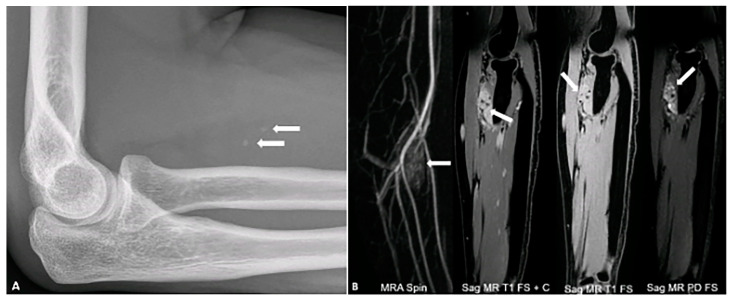
A 34-year-old female with right forearm pain. Lateral radiograph (**A**) reveals two foci of calcification (arrows) suggestive of phleboliths. Different MR sequences (**B**) show an enhancing mass (arrows) with hyperintense signal on the PD FS image, containing two internal foci of signal voids corresponding to phleboliths. The image was obtained from the University of Washington Medical Imaging database with the required permissions.

Phleboliths within a vascular tumor are almost pathognomonic for a benign hemangioma [6]. Histologically, hemangiomas represent a proliferation of blood vessels. Calcifications arise when a thrombus forms within the vasculature of the hemangioma [19]. On MR, hemangiomas appear as well-defined, lobulated masses that are isointense to muscle on T1-weighted and hyperintense on T2-weighted images, demonstrating marked contrast enhancement (Figure 4).

Angiosarcomas are aggressive malignant tumors arising from vascular endothelial cells, with a tendency for local recurrence and metastasis. The majority of angiosarcomas arise in the head and neck, most commonly the scalp, and only 10% of angiosarcomas arise in the deep soft tissues [24,25].

Vascular calcification, defined as the deposition of calcium–phosphate complexes in blood vessels, is a characteristic finding of vascular aging. Calcification at different locations may be associated with different risk factors. For example, intimal calcification is associated with atherosclerosis and increases the risk of embolic events and strokes, while medial calcification leads to arterial stiffness and reduced compliance. Vascular calcification is categorized into three types: inflammatory, metabolic, and genetic. Inflammatory type is associated with atherosclerosis, the metabolic type occurs in the media layer and is related to diabetes and chronic kidney disease, and the genetic type is linked to genetic disorders such as Marfan syndrome, typically affecting the media [26].

### 4.5. Smooth Muscle Tumors

The imaging characteristics of leiomyomas are nonspecific. They may exhibit intralesional scattered coarse or amorphous calcifications, commonly seen in degenerated uterine fibroids. In a case series, 4 of 11 cases of leiomyomas demonstrated dystrophic calcifications associated with degenerative tissue changes on histology [27]. These calcifications can also be lobulated and correlate with psammoma bodies on histology [28].

**Figure 5:**
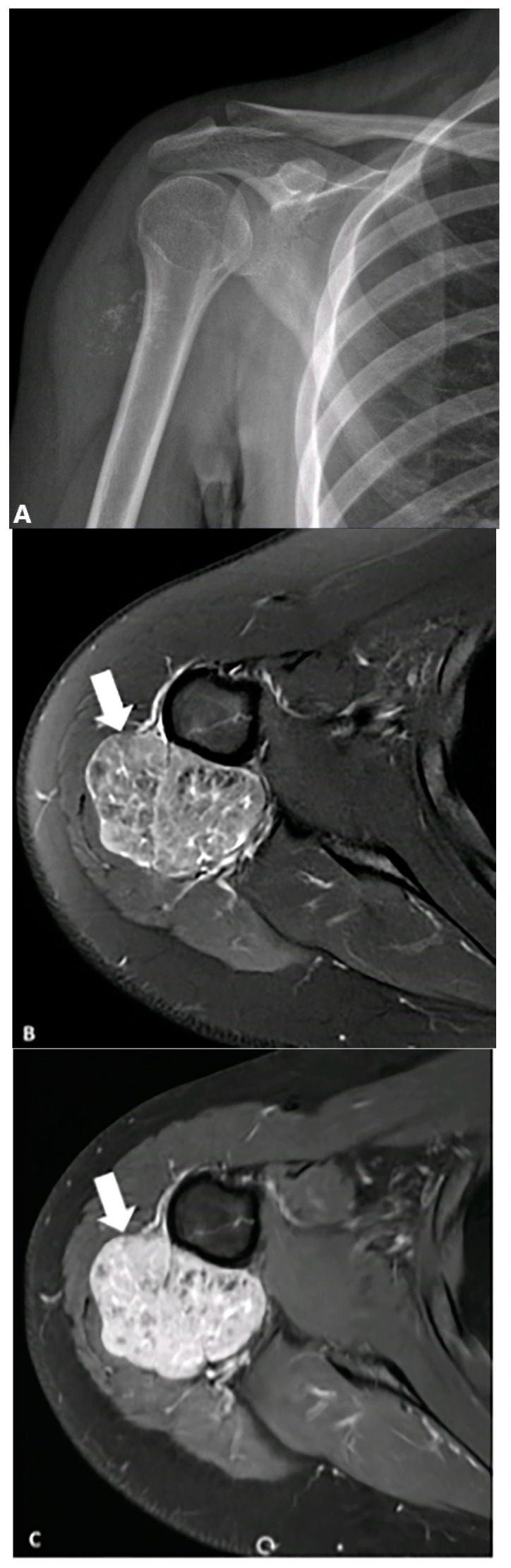
A 19-year-old female presented with a mildly tender, non-mobile mass in her right upper arm. (**A**) AP radiograph shows punctate calcifications concerning malignancy. (**B**) Axial fat-saturated T2-weighted MRI image demonstrates a heterogeneous lobulated mass (arrows) with hyperintense signal and hypointense foci corresponding to stippled (punctate) calcifications seen on the radiograph, which shows marked enhancement following contrast injection. (**C**) Biopsy confirms leiomyosarcoma. The image was obtained from the University of Washington Medical Imaging database with the required permissions.

Calcifications are neither sensitive nor specific for evaluating malignancy in smooth muscle tumors. In a study of 118 cases of leiomyosarcoma, calcifications were not identified on CT scans [29]. In two case series, radiologic evidence of calcifications was present in only 15% of cases [15,30]. When present, calcifications are usually amorphous (Figure 5) due to a dystrophic mechanism of formation [30]. On the other hand, while CT is highly sensitive for detecting calcifications, it is not infallible [31]. In some cases, calcifications may not be visualized due to their small size, low density, or obscuration by artifacts. When calcifications are not detected on CT, other imaging modalities such as gradient-echo or susceptibility-weighted MRI or dual-energy CT may provide additional insights. Dual-energy CT, in particular, can help distinguish true calcifications from other hyperdense materials such as hemorrhage or iodinated contrast [32].

### 4.6. Chondro-Osseous Tumors

**Figure 6:**
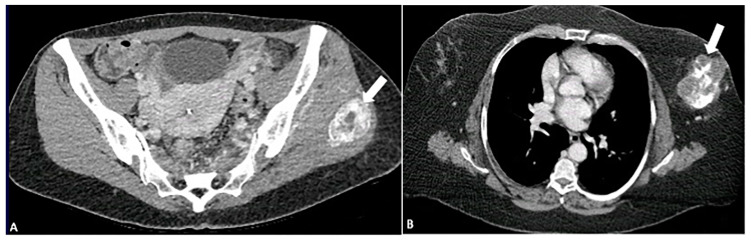
Pattern of calcification in myositis ossificans (**A**) versus osteosarcoma (**B**) on CT images. In myositis ossificans, calcifications (arrow) are organized and densest at the periphery, a phenomenon known as the “zoning phenomenon”. In contrast, osteosarcoma shows disorganized calcifications that are densest in the center (arrow). The image was obtained from the University of Washington Medical Imaging database with the required permissions.

**Figure 7:**
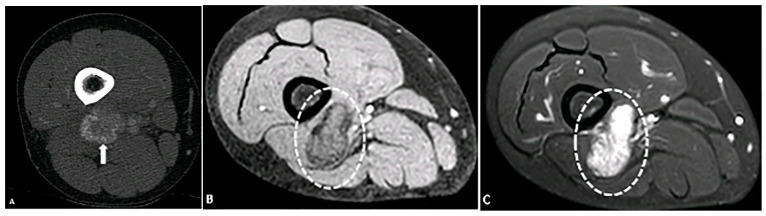
A 33-year-old female with a mass in the posterior distal aspect of the right leg. (**A**) Axial contrast-enhanced CT shows a mass with peripheral calcification (arrow). (**B**) Axial T1-weighted fat-suppressed image shows a mass (circle) with a peripheral hypointense line indicative of calcification and central hyperintensity (circle) on a T2-SPAIR fat-suppressed image (**C**). Findings are most consistent with myositis ossificans in the setting of recent trauma. The image was obtained from the University of Washington Medical Imaging database with the required permissions.

Approximately 50% of extra-skeletal osteosarcomas exhibit calcifications or osteoid matrix formation on imaging [11]. These tumors have a predilection for older adults and the lower extremities, particularly the thigh, and tend to metastasize and recur locally [33,34]. Calcifications in extra-skeletal osteosarcomas (Figure 6) are disorganized or densest in the center, as opposed to the peripheral zonal distribution of myositis ossificans (Figure 7). The tumors are usually associated with hemorrhagic and soft tissue components, which show heterogenous signal intensity on MRI. Calcification and osteoid components are hypointense and non-enhancing on MRI, often presenting as centrally located lesions.

**Figure 8:**
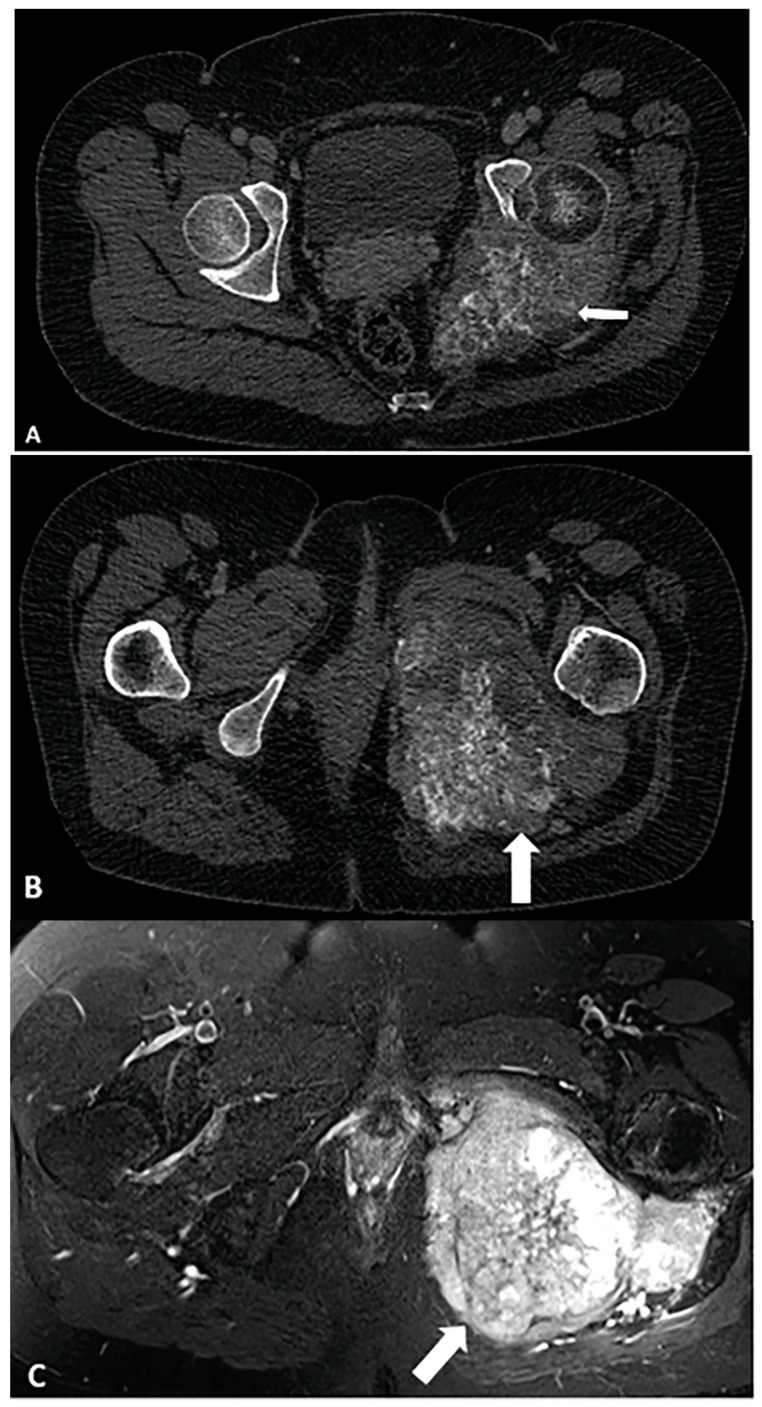
A 33-year-old female with left hip/gluteal pain. (**A**) CT demonstrates “rings and arcs” chondroid calcifications (arrow) and osseous destruction, findings most consistent with chondrosarcoma. (**B**) Contrast-enhanced CT shows enhancement in the soft tissue component of the mass (arrow). (**C**) Characteristic chondroid hyperintense signal intensity (arrow) is visible on a T2-weighted TSE fat-suppressed image. The image was obtained from the University of Washington Medical Imaging database with the required permissions.

Conventional chondrosarcomas typically present as a mixed lytic and sclerotic mass with a characteristic rig and arc pattern of calcification (Figure 8), which often allows for a confident diagnosis of cartilaginous origin. Higher-grade chondrosarcomas tend to exhibit less matrix mineralization. A more aggressive pattern of bone destruction is usually seen in less frequent subtypes, such as mesenchymal, myxoid and dedifferentiated chondrosarcomas. The characteristic matrix mineralization is typically observed on radiography and CT scans, corresponding to areas of low signal intensity on MR images [33].

### 4.7. Peripheral Nerve Sheath Tumors

Peripheral nerve sheath tumors (PNSTs) are divided into benign forms, including neurofibroma and schwannoma, and a malignant form; both types can be associated with neurofibromatosis [35].

Calcifications are exceedingly rare in malignant peripheral nerve sheath tumors. Malignant PNSTs containing calcifications were found in two cases [36,37] in the sacral region and four cases in the thorax [38], with no report in the extremities. Other imaging features can provide useful clues to assess the malignant potential of a PNST, such as the location. Benign PNSTs typically involve the cutaneous or deep nerves, whereas malignant PNSTs are more likely to affect major nerve trunks [39].

**Figure 9:**
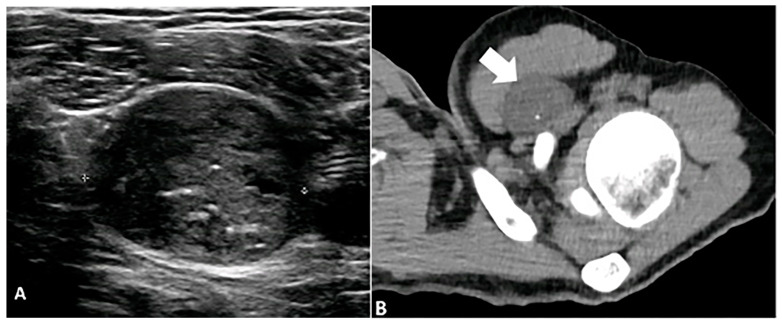
A 23-year-old male with a left subpectoral mass. (**A**) Ultrasound shows a well-circumscribed, hypoechoic soft tissue mass with internal echogenic foci, representative of punctate calcifications. The mass demonstrates water density (arrow) on CT (**B**) with punctate calcifications. Subsequent biopsy revealed a benign nerve sheath tumor, schwannoma. The image was obtained from the University of Washington Medical Imaging database with the required permissions.

Calcifications in PNSTs are most common in benign schwannomas, particularly in ancient schwannomas. This is a subtype characterized by slow growth and cystic and hemorrhagic necrotic areas [35,40,41]. These calcifications are variable in morphology, either punctate, curvilinear, or amorphous, (Figure 9) reflecting slow growth and degenerative changes [40,42]. In ancient schwannomas, degenerative cyst formation, hemorrhage, or necrosis may also be noted on MRI. Due to these features, ancient schwannomas can be mistaken for malignant tumors such as malignant PNST or undifferentiated pleomorphic sarcoma [43].

### 4.8. Tumors of Uncertain Differentiation

Synovial sarcoma commonly arises near joints, particularly in the lower extremities of young patients [44]. Synovial sarcomas show calcifications in approximately 30% of cases and should be considered in young patients with a calcified juxta-articular mass [14,45,46]. In one case series of synovial sarcomas, all 17 calcified cases demonstrated a stippled morphology, either scattered or conglomerates [45]. In another case series, all six cases of calcified synovial sarcoma demonstrated punctate peripheral calcifications [47]. However, amorphous and coarse calcifications were also reported [14]. The Triple sign on MRI, characterized by low signal intensity of dystrophic calcification and fibrotic bands, intermediate signal intensity of soft tissue, and high signal intensity of necrosis, is characteristic. Since synovial sarcomas often contain calcifications, their presence should raise suspicion and prompt consideration for biopsy. This is especially important as synovial sarcomas can sometimes mimic benign lesions by exhibiting slow growth, a homogeneous appearance, and well-defined margins [2]. Aggressive early management is indicated to reduce the local recurrence and metastatic spread [14,46].

Undifferentiated pleomorphic sarcoma is a common type of soft tissue sarcoma with a high rate of local recurrence and metastasis. Their imaging features can vary widely depending on cellularity and the presence of hemorrhage and necrosis. While calcification is not a specific finding of this tumor, it can be observed in 5–20% of cases. These calcifications are punctate and curvilinear with a peripheral predilection [1], although coarse and amorphous calcifications are also reported [48,49]. Undifferentiated pleomorphic sarcoma is typically a heterogenous soft tissue mass and may demonstrate peripheral enhancement when central hemorrhage and necrosis are present [1,50].

## 5. Non-Neoplastic Soft Tissue Calcifications

### 5.1. Calcific Tendinitis

Calcific tendinitis refers to the deposition of calcium hydroxyapatite within tendons. It most commonly happens in the shoulders (rotator cuff) causing joint pain. However, it can also occur in other anatomical sites, including the elbows (common extensor tendon), wrists (flexor carpi ulnaris), knees (quadriceps and patellar tendons), and ankles (Achilles tendon, peroneal tendons) [51]. While less frequent in these locations, the underlying pathophysiology remains similar. Calcifications in calcific tendinitis are typically amorphous [51]. However, their appearance can vary from amorphous to dense, and from well-defined to ill-defined shapes, depending on the stage of development. Calcific tendinitis is related to localized hypoxia leading to fibrocartilaginous metaplasia, where tenocytes transform into chondrocytes [52]. When calcifications are noted around tendon insertion sites, calcific tendinitis should be considered.

### 5.2. Metabolic Disease

#### 5.2.1. Gout

Gout is a metabolic disorder caused by the deposition of monosodium urate crystals in the articular and periarticular soft tissues resulting in inflammatory arthritis. Radiological findings of Gout depend on the stage of the disease. Soft tissue calcifications happen in advanced and chronic cases. In chronic gout, tophi are seen as dense soft-tissue nodules in the periarticular soft tissues or within the bursae with or without amorphous calcifications. Juxta-articular erosions are usually seen adjacent to tophi, as they frequently represent an intraosseous extension of tophi [53].

The characteristic ultrasonographic “double contour sign” occurs when MSU crystals precipitate on the surface of the hyaline cartilage. Tophi present as circumscribed, inhomogeneous, hyperechoic, and/or hypoechoic aggregations often surrounded by a small anechoic rim [54].

#### 5.2.2. Primary Tumoral Calcinosis

Primary tumoral calcinosis is a rare hereditary metabolic disease associated with phosphate regulation dysfunction. It has calcium phosphate crystals deposited in peri-articular soft tissue, most commonly the hip and shoulder [55,56]. Although pathogenesis has not been fully understood, mutations in genes such as GalNAc transferase 3 (GALNT3), FGF23, and KLOTHO cause hyperphosphatemia, causing calcific deposits to fill neo-bursae created by dysfunctional histiocytes [55,57]. Radiographs typically show amorphous, multilobulated calcifications around joints. On CT, these calcified masses can contain fluid-calcium levels [56]. On MRI, these calcifications manifest as multiple lobular non-enhancing hypointense lesions on both T1- and T2-weighted images, sometimes with osseous erosions [43,56].

Tumoral calcinosis associated with chronic renal failure is a primary differential consideration for a lobular periarticular calcified mass. The pathogenesis of calcium deposition and imaging findings are similar to those of primary tumoral calcinosis [55]. Like primary tumoral calcinosis, calcific deposits in chronic renal failure are often bilateral, multiple, and mass-like. It can occur in up to 7% of patients on hemodialysis, with the average time after starting hemodialysis varying from a few months to several years [58].

**Figure 10:**
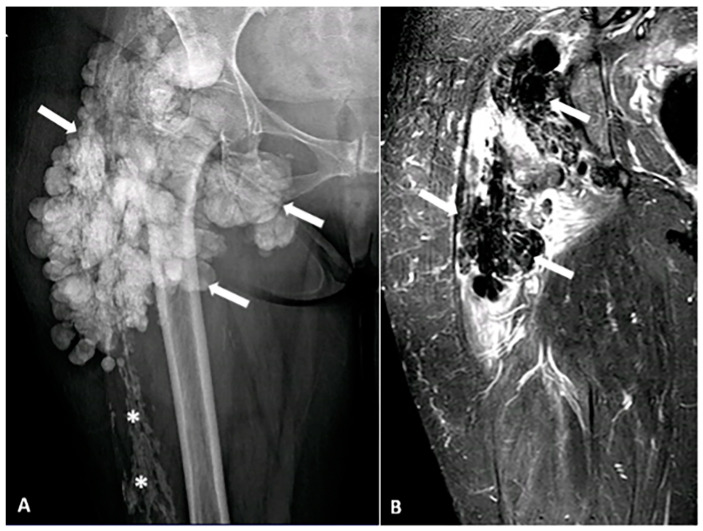
A 52-year-old female with a firm, palpable mass in the right hip. She also has sclerodactyly, dysphagia, Raynaud’s phenomenon, and polyarthralgia. (**A**) Anteroposterior (AP) radiograph of the femur shows clustered, lobulated calcifications (arrows) consistent with pseudotumoral calcinosis, which can be associated with connective tissue diseases. Sheet-like calcifications (star) indicating calcinosis cutis, commonly associated with systemic sclerosis. (**B**) Coronal MR STIR image demonstrates confluent areas of signal voids, corresponding to foci of calcification. The image was obtained from the University of Washington Medical Imaging database with the required permissions.

The prevalence of calcinosis in systemic sclerosis has been reported to range from 18% to 49%. It is more frequently described in the limited cutaneous subset, though patients with the diffuse form are also commonly affected. Pathogenesis includes mechanical stress, tissue hypoxia, and insufficient blood flow. Pseudotumoral calcinosis is most commonly found in the hands and wrists, but the shoulder and hips (Figure 10) can be also involved, with a typically symmetrical distribution. Most patients report functional disability and pain, with some cases leading to possible ulceration and superinfection [56,57].

### 5.3. Pilomatricoma

Pilomatrixoma (PM), also known as calcifying epithelioma of Malherbe (CEM), is a benign tumor originating from hair follicle matrix in superficial subcutaneous tissue. It usually grows slowly, and malignant transformation into pilomatrix carcinoma is exceedingly rare [59].

Differentiating PM from epidermoid cysts is crucial, as pilomatrixomas may require complete surgical excision to prevent recurrence [60]. Intramural calcification is a characteristic feature of PM. It is often visualized as a heterogeneous tumor with internal echogenic foci and a hypoechoic rim or a completely echogenic mass with strong posterior acoustic shadowing in the subcutaneous layer [61].

### 5.4. Synovial Chondromatosis

**Figure 11:**
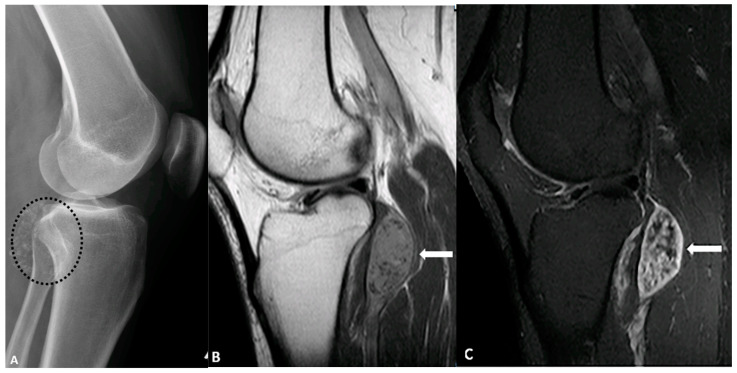
A 43-year-old female with left knee pain. (**A**) Lateral radiograph of the knee demonstrates multiple foci of calcification (circle) adjacent to the knee joint. (**B**) Sagittal T1-weighted pre- and post-contrast MRI (**B**,**C**) reveals isointense intra-articular enhancing nodules (arrows) containing non-enhancing hypointense foci, corresponding to the areas of calcification. These findings are consistent with synovial chondromatosis. The image was obtained from the University of Washington Medical Imaging database with the required permissions.

Synovial chondromatosis is a benign proliferative process of the synovium. It is characterized by multiple intra-articular chondroid nodules demonstrating “ring-and-arc” and punctate mineralization, most commonly in the knee and hip joints [62]. Calcifications are radiographically evident in approximately 70–95% of cases [62,63]. Synovial chondromatosis can rarely transform into chondrosarcoma. Osseous erosion can be seen in 20–50% of patients with synovial chondromatosis [64,65]. On CT, calcifications are associated with lobular synovial thickening, which is hypoattenuating due to fluid content. On the other hand, on MR, synovial chondromatosis appears as lobular T1 isointense (compared to muscle), T2 hyperintense enhancing nodules (Figure 11) that contain non-enhancing hypointense foci, representing mineralization [62].

### 5.5. Calcific Myonecrosis

**Figure 12:**
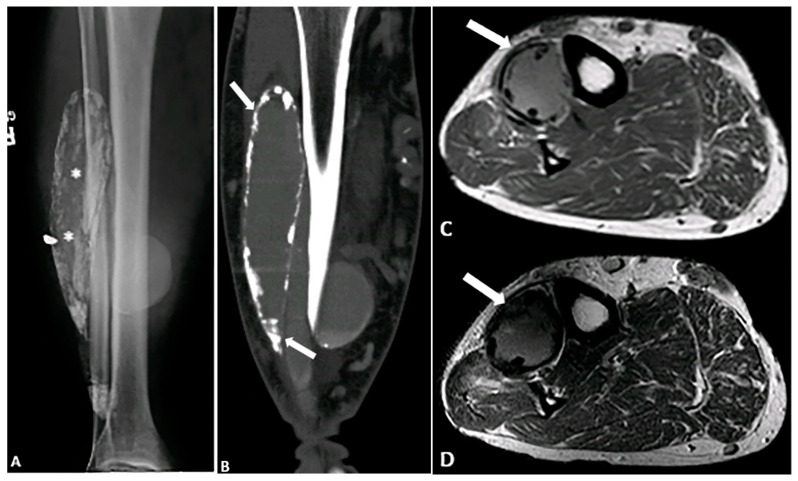
A 71-year-old male with a remote history of traumatic injury to the femoral artery and multiple fasciotomies. (**A**,**B**) Radiograph and CT demonstrate a calcified fusiform mass within the anterior compartment of the lower leg, with multiple foci of calcification within the mass and in its periphery (* and arrow). (**C**,**D**) Axial T1-weighted (**C**) and T2-weighted SPAIR (**D**) images reveal an intermediate signal intensity mass (arrows) with peripheral susceptibility artifacts due to calcium deposition. These findings are compatible with calcific myonecrosis. The image was obtained from the University of Washington Medical Imaging database with the required permissions.

Calcific myonecrosis manifests as a fusiform mass with sheet-like calcifications in the periphery of a muscle or muscular compartment. It most commonly affects the anterior compartment of the lower extremity. These masses can contain fluid-calcium levels and can cause smooth osseous erosion due to chronic pressure. As its name suggests, calcific myonecrosis is secondary to post-traumatic ischemia and cystic necrosis of muscle. On MR, calcific myonecrosis typically demonstrates cystic liquefactive components (Figure 12) with homogenous intermediate T1 signal and heterogenous high T2 signal [66].

### 5.6. Injection Granuloma

Injection granulomas are small soft tissue nodules with dystrophic calcifications that form as a localized inflammatory response to iatrogenic injections. As such, these nodules commonly occur within the subcutaneous fat of the gluteal regions and ventral abdominal wall. On MR, these nodules are typically hypointense on T1-weighted images with variable signal intensity on T2-weighted images. There can be a surrounding high T2 signal intensity if acute inflammation is present [67].

### 5.7. Cysticercosis

Cysticercosis, a disease caused by Taenia Solium infection, can cause small ovoid calcifications in the subcutaneous tissue and muscle. It represents a calcified cyst containing dead larvae, which have triggered a granulomatous reaction [68]. Numerous calcifications are typically present, often described as “millet seed” or “rice grain” shaped. Some radiologists compare this pattern to a “starry sky” appearance [69,70]. On MR, intramuscular cysts are typically T1 hypointense and T2 hyperintense. There is peripheral edema and enhancement in the inflammatory stages preceding the formation of granulomatous calcifications [69].

### 5.8. Calcinosis Cutis

Calcinosis cutis is categorized as dystrophic calcium deposition in connective tissue, as the soft tissues in connective tissue disorders are prone to calcification despite normal blood calcium and phosphorus levels. This is likely due to chronic inflammation and hypoxia. The discovery of subcutaneous dystrophic calcification often warrants further investigation for underlying connective tissue diseases (Figure 10) such as mixed connective tissue disease, dermatomyositis, and progressive systemic sclerosis [71].

The term Calcinosis cutis universalis is used when there are extensive plaques or sheet-like calcifications which can be seen on radiographs in various connective tissue diseases. The designation “universalis” applies to widespread superficial soft tissue calcification. In contrast, calcinosis cutis circumscripta refers to a localized form that often affects the hands and feet [71].

## 6. Future Directions in Imaging

Future advancements in imaging modalities and treatment strategies are expanding soft tissue tumors’ diagnostic and therapeutic landscapes. The evolution of imaging technologies and artificial intelligence is poised to significantly enhance the detection and characterization of calcifications [72,73]. By leveraging radiomics and machine learning algorithms, AI-driven approaches can extract subtle imaging features that may escape human perception, thereby improving diagnostic precision [74]. These modalities enhance contrast resolution and provide detailed tissue characterization, potentially aiding in the early detection of tumors and the differentiation of neoplastic and non-neoplastic calcifications.

## 7. Conclusions

Imaging diagnosis of soft tissue tumors remains challenging in musculoskeletal radiology due to the diversity of soft tissue tumors and the significant overlap in their imaging features. Amid these challenges, calcifications can be a vital clue for diagnosis and management planning. While an imaging-based diagnosis based on tumor characterization is valuable, there are cases where the diagnosis remains ambiguous. If there is uncertainty about malignancy, biopsy should be performed.

Familiarity with typical non-neoplastic soft tissue calcification is also important in the differential diagnosis of intratumor and soft tissue calcifications.

## Figures and Tables

**Table 1:** WHO classification of soft tissue tumors containing calcifications.

Category of Soft Tissue Tumor	Benign	Malignant
Adipocytic		Dedifferentiated, Well-differentiated liposarcoma
Fibroblastic/Myofibroblastic	Myositis ossificans, Calcifying aponeurotic fibroma	
Fibrohistiocytic		
Vascular	Hemangioma	
Pericytic		
Smooth muscle	Leiomyoma	
Skeletal muscle		
Gastrointestinal stromal		
Chondro-osseous	Soft tissue chondroma	Extra-skeletal osteosarcoma
Peripheral nerve sheath	Ancient schwannoma	
Uncertain differentiation		Synovial sarcoma, Undifferentiated pleomorphic sarcoma
Undifferentiated small round cell sarcoma		

**Table 2:** Non-neoplastic soft tissue calcifications.

Disease	Characteristics
Calcific tendinitis	Tendon insertion sites
Gout	Pain, specific area (e.g., first toe), obese man, hyperuricemia
Tumoral calcinosis a/w metabolism	Periarticular calcified mass
Pilomatricoma	A superficial well-demarcated dense calcification
Synovial chondromatosis	Intra-articular homogeneous round calcifications
Calcific myonecrosis	Traumatic history, necrotic mass with peripheral calcifications
Injection granuloma	Gluteal subcutaneous fat
Cysticercosis	Diffuse rice-grain calcifications
Calcinosis cutis	Superficial calcifications in systemic sclerosis

## Data Availability

No new data were created or analyzed in this study. Data sharing is not applicable to this article.
